# Enhancing rapeseed drought resilience: transcriptomic insights from superabsorbent polymer seed coatings

**DOI:** 10.1186/s13104-026-07750-5

**Published:** 2026-02-27

**Authors:** Akram Abdolmaleki, Hendrik Bertram, Susann Michanski, Armin O. Schmitt, Mehmet Gültas

**Affiliations:** 1 Georg-August University, Margarethe Von Wrangell-Weg 7, 37075 Göttingen, Germany; 2 South Westphalia University of Applied Sciences, Lübecker Ring 2, 59494 Soest, Germany

**Keywords:** *Brassica napus* L., Rapeseed, mRNA seq, SAP, Transcriptome analysis, Drought stress, Seed Germination

## Abstract

**Objectives:**

Climate change leads to drought that critically threatens global food security by limiting plant growth and crop productivity. Rapeseed, a vital source of feed, food, and oil, is highly vulnerable to this stress. To mitigate this, novel agricultural materials such as superabsorbent polymers (SAPs) offer a promising short-term solution to alleviate drought stress. Furthermore, understanding how these polymers enhance drought tolerance can help identify candidate genes for long-term breeding programs. Notably, this study is the first comprehensive investigation to evaluate SAP effects at both molecular and biological levels using mRNA sequencing, providing significant insights into their drought-mitigating mechanisms in rapeseed.

**Data description:**

Messenger RNA sequencing (mRNA-seq) was conducted on five groups: two fossil-based polymers (MERCK, SWT), one natural-based polymer (ABG), a stress control (CS), and a well-watered control (CN), to assess induced transcriptomic changes. Following the International Seed Testing Association guidelines, the experiment was conducted on rapeseed for 7 days (Rajendra Prasad in Seed science and technology: biology, production, quality, Springer Nature Singapore, 2023). Afterwards, indexed cDNA libraries were prepared for mRNA sequencing using the Illumina NovaSeq X Plus platform. For this study, we collected 10 mRNA sequencing samples, distributed across the different treatment groups. The resulting FASTQ files from sequencing are publicly available in the European Nucleotide Archive under accession number ERP180393.

## Objective

Rapeseed, a key crop for global food and oil production, is highly vulnerable to drought, a major abiotic stress that restricts its cultivation. Water deficits impair every stage of its life cycle, reducing vegetative growth, biomass accumulation, reproductive development, and final yield [2, 3]. The germination and seedling stages are particularly critical, as they exhibit elevated sensitivity to drought and lay the foundation for successful establishment [4]. During this early phase, drought stress significantly reduces germination rates, potential, and indices, while inhibiting plumule and radicle growth, ultimately compromising crop success [4, 5]. Given these critical early-stage vulnerabilities and the increasing drought intensity driven by climate change, strategies to enhance resilience in this crop are urgently needed [6].

One promising approach to coping with drought stress in agriculture involves utilizing innovative materials such as SAPs (7). SAPs, characterized as three-dimensional hydrophilic polymers with a cross-linked network structure, exhibit the remarkable ability to absorb water hundreds to thousands of times their weight [8–10]. While extensive efforts have explored drought stress responses in this important plant, most previous studies have focused on specific phenotypic traits, such as the effects of SAPs on physiological traits[11–15]. Therefore, a more comprehensive study, integrating molecular and phenotypic analyses, is required to not only fully elucidate the effects of diverse SAPs on key plant growth pathways but also to compare the environmental footprint and degradability of natural versus fossil-based products. This investigation provides a unique dataset, shedding light on the intricate mechanisms by which different SAP types enhance rapeseed drought resilience, offering valuable insights for both sustainable agricultural strategies and the development of environmentally sustainable solutions.

### Data description

This data note presents mRNA-seq datasets from rapeseed seedlings treated with SAPs under drought conditions for 7 days.

The data, comprising 10 raw FASTQ files of mRNA reads, cover five distinct treatment groups for each species: MERCK, SWT, ABG, CS, and CN. All files are deposited in ENA (http://identifiers.org/ena.embl:PRJEB97951).

Seeds were coated with the selected SAPs following the manufacturer’s instructions, with adjustments made for seed size and absorption capacity.

The experimental material consisted of winter rapeseed (*Brassica napus* L. *subsp. napus var. napus f. biennis Thell.*, accession CR 3261, Germany), provided by the Leibniz Institute of Plant Genetics and Crop Plant Research (IPK). To evaluate the impact of superabsorbent polymers (SAPs) on germination and gene expression under moisture deficit, five treatments were established: three SAP-coated groups (Merck, SWT, and ABG) and two uncoated control groups: a well-watered control (CN) and a drought-stress control (CS). SAP application rates were determined by normalizing polymer quantities to ensure equivalent water availability per seed across all treatments, accounting for the thousand-seed weight (TSW = 3.5 g), specific water-absorption capacities (WAC), manufacturer recommendations, and preliminary optimization tests. Consequently, the fossil-based polymers Merck (MKCR9032, Germany) and SWT (Isonem, Turkey) were applied at 28.5 g/kg and 57.0 g/kg of seed, respectively, while the natural-based polymer ABG (AgroBiogel GmbH, Austria) was applied at 1129 g/kg. Following seed treatment, a germination assay was conducted in which 10 seeds per replicate were placed on a double layer of filter paper (grade 3 hw, diameter 90 mm, Göttingen, Germany) in Petri dishes. Drought stress was initiated at the time of seed placement by applying a restricted water volume of 2 mL to the CS and SAP-treated groups, while the CN group received 5 mL to maintain optimal moisture. These specific water volumes were determined based on preliminary optimization trials and established protocols [1] to ensure a significant yet non-lethal environmental constraint throughout the seven days. Ten replicates per treatment were incubated in a climate-controlled chamber (BINDER KBW 720, Tuttlingen, Germany) at 25 ± 1 °C under a 16/8 h light/dark cycle.

Total RNA was extracted from seedling tissues using the TIANGEN RNAprep Pure Plant Kit with DNase I digestion, and RNA integrity was confirmed with an Agilent 2100 Bioanalyzer. Messenger RNA was enriched with oligo(dT) magnetic beads, fragmented, and converted into strand-specific cDNA libraries. Libraries were subjected to end repair, A-tailing, adapter ligation, amplification, and size selection. Their concentration and integrity were assessed with Qubit fluorometry, real-time PCR, and Bioanalyzer assays. All laboratory procedures were carried out by Novogene (Munich, Germany). Sequencing was performed on an Illumina NovaSeq X Plus platform using paired-end mode. The final dataset comprises 16 quality-controlled FASTQ files, with each of the five treatments represented. The data are publicly available in ENA under ERP180393. An overview of the datasets is provided in Table 1.

**Table 1:** Overview of mRNA-seq FASTQ datasets from rapeseed seedlings subjected to seed coating with SAPs under drought stress

Label	Name of data file/data set	File types (file extension)	Data repository and identifier (DOI or accession number)
Data file 1	RCN602	FASTQ (.fastq.gz)	http://identifiers.org/ena.embl:ERR15607436 [16]
Data file 2	RCN603	FASTQ (.fastq.gz)	http://identifiers.org/ena.embl:ERR15606935 [17]
Data file 3	RCS701	FASTQ (.fastq.gz)	http://identifiers.org/ena.embl:ERR15607438 [18]
Data file 4	RCS702	FASTQ (.fastq.gz)	http://identifiers.org/ena.embl:ERR15607437 [19]
Data file 5	RMERCK1002_1	FASTQ (.fastq.gz)	http://identifiers.org/ena.embl:ERR15607442 [20]
Data file 6	RMERCK1002_2	FASTQ (.fastq.gz)	http://identifiers.org/ena.embl:ERR15607441 [21]
Data file 7	RMERCK1003_1	FASTQ (.fastq.gz)	http://identifiers.org/ena.embl:ERR15607439 [22]
Data file 8	RMERCK1003_2	FASTQ (.fastq.gz)	http://identifiers.org/ena.embl:ERR15607440 [23]
Data file 9	RSWT802_1	FASTQ (.fastq.gz)	http://identifiers.org/ena.embl:ERR15607443 [24]
Data file 10	RSWT802_2	FASTQ (.fastq.gz)	http://identifiers.org/ena.embl:ERR15607444 [25]
Data file 11	RSWT801_1	FASTQ (.fastq.gz)	http://identifiers.org/ena.embl:ERR15607445 [26]
Data file 12	RSWT801_2	FASTQ (.fastq.gz)	http://identifiers.org/ena.embl:ERR15607446 [27]
Data file 13	RABG903_1	FASTQ (.fastq.gz)	http://identifiers.org/ena.embl:ERR15606928 [28]
Data file 14	RABG903_2	FASTQ (.fastq.gz)	http://identifiers.org/ena.embl:ERR15606931 [29]
Data file 15	RABG901_1	FASTQ (.fastq.gz)	http://identifiers.org/ena.embl:ERR15606933 [30]
Data file 16	RABG901_2	FASTQ (.fastq.gz)	http://identifiers.org/ena.embl:ERR15606934 [31]

*File names follow the format: [Crop][Treatment][ReplicateID]_[LaneSplit]. Note: All Merck, SWT, ABG, and CS samples were subjected to drought stress; only CN samples were maintained under well-watered (unstressed) conditions. Files with suffixes _1 and _2 represent technical lane splits of the same biological replicate and were merged for downstream analysis.

## Limitations

This study offers valuable initial insights into the molecular responses of plants to SAP application during drought stress. To further advance our understanding, several key areas warrant future exploration:

Primarily, while the present experimental design, which employs two biological replicates per treatment group (given foundational constraints), effectively revealed robust transcriptional shifts, increasing the number of replicates in subsequent investigations would significantly boost statistical power. This enhancement would enable the detection of more subtle or variable gene regulatory patterns, thus improving the overall reliability and resolution of differential gene expression analyses. Secondarily, drawing from prior research that underscored variety-specific responses to SAPs [32], our current investigation concentrated on a single rapeseed variety. Future studies would greatly benefit from integrating a wider array of varieties. This approach is essential for evaluating the generalizability of the observed molecular adaptations across diverse genetic backgrounds and for pinpointing variety-specific responses to SAP application under drought stress.

## Data Availability

The data described in this Data note can be freely and openly accessed in the European Nucleotide Archive (ENA ) under ERP180393 (Table 1).

## Abbreviations

SAP: Super abandonment polymer
SWT: Isonem soil water trap
ABG: AgroBioGel
CS: Control stress
CN: Control normal
ISTA: International Seed Testing Association
ENA: The European Nucleotide Archive
mRNA-Seq: mRNA sequencing
